# Association of ketamine use with lower risks of post-intubation hypotension in hemodynamically-unstable patients in the emergency department

**DOI:** 10.1038/s41598-019-53360-6

**Published:** 2019-11-21

**Authors:** Tadayoshi Ishimaru, Tadahiro Goto, Jin Takahashi, Hiroshi Okamoto, Yusuke Hagiwara, Hiroko Watase, Kohei Hasegawa, Emergency Network Investigators, Hiroshi Morita, Takahisa Kawano, Yohei Kamikawa, Hideya Nagai, Takashi Matsumoto, Suguru Nonami, Yusuke Miyoshi, Sho Segawa, Yuya Kitai, Kenzo Tanaka, Saburo Minami, Hiromasa Yakushiji, Hiroshi Okamoto, Naoto Miyauchi, Yukari Goto, Nobuhiro Sato, Koichiro Gibo, Masashi Okubo, Yukiko Nakayama, Nobuhiro Miyamae, Hirose Kaoru, Taichi Imamura, Azusa Uendan, Yasuaki Koyama, Hiroshi Kamura, Nakashima Yoshiyuki, Jin Takahashi, Jin Irie, Nobunaga Okada, Seiro Oya, Akihiko Inoue

**Affiliations:** 1 Department of Emergency and Critical Care Medicine, Tokyo Bay Urayasu Ichikawa Medical Center, 3-4-32 Todaijima, Urayasu, Chiba 279-0001 Japan; 2 Graduate School of Medical Sciences, University of Fukui, 23-3, Shimoaizuki, Matsuoka, Eiheiji, Yoshida, Fukui 910-1193 Japan; 3 Department of Critical Care Medicine, Kurashiki Central Hospital, 1-1-1 Miwa, Kurashiki, Okayama 710-8602 Japan; 4 Department of Pediatric Emergency and Critical Care Medicine, Tokyo Metropolitan Children’s Medical Center, 2-8-29 Musashidai, Fuchu, Tokyo 183-8561 Japan; 5 Department of Radiology, University of Washington, 850 Republican Street Seattle, Washington, WA 98006 USA; 6 Department of Emergency Medicine, Massachusetts General Hospital, Harvard Medical School, Boston, MA USA; 7 Fukui University Hospital, Fukui, Japan; 8 Fukui Prefectural Hospital, Fukui, Japan; 9 Kameda Medical Center, Chiba, Japan; 10 Kishiwada Tokushukai Hospital, Osaka, Japan; 11 Nagoya Ekisaikai Hospital, Nagoya, Japan; 12 Niigata City General Hospital, Niigata, Japan; 13 Okinawa Chubu Prefectural Hospital, Okinawa, Japan; 14 Otowa Hospital, Kyoto, Japan; 15 Shonan Kamakura General Hospital, Kanagawa, Japan; 16 St. Marianna University School of Medicine Hospital, Kanagawa, Japan; 17 Tokyo Bay Urayasu Ichikawa Medical Center, Chiba, Japan; 18 University Hospital, Kyoto Prefectural University of Medicine, Kyoto, Japan; 19 Yokohama Rosai Hospital, Kanagawa, Japan; 20 Hyogo Emergency Medical Center, Hyogo, Japan

**Keywords:** Drug development, Fluid dynamics

## Abstract

To determine whether ketamine use for tracheal intubation, compared to other sedative use, is associated with a lower risk of post-intubation hypotension in hemodynamically-unstable patients in the emergency department (ED), we analyzed the data of a prospective, multicenter, observational study—the second Japanese Emergency Airway Network (JEAN-2) Study—from February 2012 through November 2017. The current analysis included adult non-cardiac-arrest ED patients with a pre-intubation shock index of ≥0.9. The primary exposure was ketamine use as a sedative for intubation, with midazolam or propofol use as the reference. The primary outcome was post-intubation hypotension. A total of 977 patients was included in the current analysis. Overall, 24% of patients developed post-intubation hypotension. The ketamine group had a lower risk of post-intubation hypotension compared to the reference group (15% vs 29%, unadjusted odds ratio [OR] 0.45 [95% CI 0.31–0.66] p < 0.001). This association remained significant in the multivariable analysis (adjusted OR 0.43 [95% CI 0.28–0.64] p < 0.001). Likewise, in the propensity-score matching analysis, the patients with ketamine use also had a significantly lower risk of post-intubation hypotension (OR 0.47 [95% CI, 0.31–0.71] P < 0.001). Our observations support ketamine use as a safe sedative agent for intubation in hemodynamically-unstable patients in the ED.

## Introduction

Tracheal intubation for hemodynamically-unstable patients is a critical resuscitation procedure in the emergency department (ED). Yet, the literature has documented that the adverse event rate in ED patients who underwent airway management remains high—e.g., 22% with post-intubation hypotension and up to 4% with cardiac arrest^1,2^. Building strong evidence base for intubation medications (e.g., sedative agents) is critical for the development of optimal intubation strategies in hemodynamically-unstable patients (e.g., patients with pre-intubation hypotension and shock) who are at high risk for these clinically-important adverse events^3^.

Ketamine—a sympathomimetic agent that releases catecholamines and inhibits their reuptake^4^ —has been recommended for patients at high risk for hypotension and cardiac arrest^5^, albeit the limited evidence. Within the sparse literature, a randomized controlled trial of 80 operating room patients has demonstrated that ketamine increased the mean arterial pressure by 10% during induction^6^. In contrast, other small-scale studies (n ≤ 112) have also reported that ketamine use was associated with blunted hypertensive responses and post-intubation hypotension in hemodynamically-unstable patients^7,8^. Despite the clinical importance, there remains a controversy over the relationship of ketamine use with the risk of post-intubation hypotension in the ED.

To address the knowledge gap in the literature, we analyzed the data from a large multicenter prospective study to determine the association of ketamine use—compared with midazolam or propofol use—with the risk of post-intubation hypotension in hemodynamically-unstable ED patients.

## Results

During the 46-month study period, the second Japanese Emergency Airway Network (JEAN-2) recorded a total of 7,657 patients with emergency airway management in the 15 EDs (capture rate, 97%; Supplemental Fig. 1). We excluded 3,183 patients with cardiac arrest, 169 children (aged <18 years), 2,361 patients with a shock index (SI) of <0.9, 511 patients without sedative use, 50 patients intubated with other sedatives, 18 patients intubated with multiple sedatives, and 388 patients with missing data. The remaining 977 patients were eligible for the current analysis. Overall, the median age was 67 years (interquartile range, 53–78 years) and 68% were male. Ketamine was used in 316 patients (32%). The baseline characteristics of the ketamine and reference groups are summarized in Table 1. The ketamine group was more likely to be intubated for shock (53%) and by an emergency medicine resident, compared to the reference group (both P < 0.05).

**Table 1:** Baseline characteristics and post-intubation hypotension in hemodynamically unstable patients in the emergency department, according to ketamine use

	Ketamine group n = 316 (32%)	Reference group n = 661 (68%)	P-value
**Patient characteristics**			
Age, median (IQR), years	69 (55–79)	67 (52–77)	0.14
Male sex	209 (66)	460 (70)	0.28
**Body mass index** (**kg/m**^2^**)**			
<18.5	63 (20)	111 (17)	0.23
18.5–24.9	169 (53)	398 (60)	0.046
≥25.0	84 (27)	152 (23)	0.22
**Airway management characteristics**			
Primary indication*			
Respiratory failure	75 (24)	246 (37)	<0.001
Medical shock	166 (53)	144 (22)	<0.001
Traumatic indication	40 (13)	98 (15)	0.36
Others^†^	35 (11)	173 (26)	<0.001
Premedication use	111 (35)	216 (33)	0.45
Neuromuscular blocker use^‡^	254 (80)	487 (74)	0.02
**Specialty of intubator**			
Transitional-year resident^§^	92 (29)	244 (37)	0.02
Emergency medicine resident	136 (43)	224 (34)	0.004
Emergency physician	51 (16)	121 (18)	0.41
Other specialties	37 (12)	72 (11)	0.70
**Outcome event**			
Post-intubation hypotension^||^	47 (15)	180 (27)	<0.001

Abbreviation: IQR, interquartile range.

Data are shown as n (%) unless otherwise specified.

*Percentages may not equal 100 due to rounding.

^†^Defined as airway obstruction, altered mental status, and other medical indications.

^‡^With or without succinylcholine, rocuronium, or vecuronium.

^§^Defined as post-graduate years 1 or 2.

^||^Systolic blood pressure of ≤90 mmHg during the 30-minute period following intubation or ≥20% decrease in systolic blood pressure between pre-intubation and immediately after intubation.

**Figure 1:**
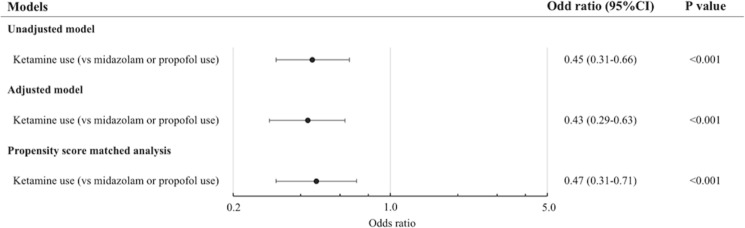
The association of ketamine use of post-intubation hypotension in unadjusted and adjusted models, and propensity score matched analysis. Compared with the reference group, ketamine use was significantly associated with a lower risk of post-intubation hypotension in both unadjusted and adjusted models. This association was consistent in the propensity score matching analysis.

Overall, the incidence of post-intubation hypotension (i.e., systolic blood pressure [SBP] of ≤90 mmHg or ≥20% decrease in SBP) was 23%. The ketamine use was associated with a significantly lower risk of post-intubation hypotension compared with the reference group (15% vs. 27%; unadjusted odds ratio [OR], 0.45; 95% CI, 0.31–0.66; P < 0.001; Table 2). This association remained significant in the multivariable model adjusting for potential confounders (adjusted OR, 0.43; 95% CI, 0.29–0.65; P < 0.001; Fig. 1).

**Table 2:** Unadjusted and adjusted associations of ketamine use with post-intubation hypotension in hemodynamically unstable patients in the emergency department.

Model and covariate	Odds ratio (95% CI)	P-value
**Unadjusted model**		
Ketamine use (vs midazolam or propofol use)	0.45 (0.31–0.66)	<0.001
**Adjusted model**		
Ketamine use (vs midazolam or propofol use)	0.43 (0.29–0.65)	<0.001
***Covariates***		
Age (per each incremental year)	1.02 (1.01–1.03)	<0.001
Male sex	0.71 (0.50–0.99)	0.04
**Body mass index (kg/m**^2^**)**		
<18.5	Reference	
18.5–24.9	0.92 (0.60–1.41)	0.71
≥25.0	1.17 (0.72–1.91)	0.54
**Primary indication**		
Respiratory failure	Reference	
Medical shock	0.64 (0.43–0.96)	0.03
Traumatic indication	0.61 (0.35–1.06)	0.08
Others*	0.41 (0.25–0.66)	<0.001
Premedication use	1.66 (1.09–2.52)	0.02
Neuromuscular blocker use^†^	1.00 (0.68–1.49)	0.99
**Specialty of intubator**		
Transitional-year resident^‡^	Reference	
Emergency medicine resident	0.90 (0.48–1.69)	0.74
Emergency physician	0.81 (0.50–1.29)	0.37
Other specialties	1.00 (0.60–1.66)	0.99

Abbreviation: CI, confidence interval.

*Defined as airway obstruction, altered mental status, and other medical indications.

^†^With or without succinylcholine, rocuronium, or vecuronium.

^‡^Defined as post-graduate years 1 or 2.

In the sensitivity analysis, using one-to-one propensity score matching, the baseline characteristics of 286 matched pairs were successfully balanced between the ketamine and reference groups (all standardized differences of <10; Supplemental Table 1). Similar to the primary analysis, the patients with ketamine use had a significantly lower risk of post-intubation hypotension, compared to the patients in the reference group (16% vs. 28%), with a corresponding OR of 0.47 (95% CI, 0.31–0.71; P < 0.001; Fig. 1). In the analysis stratified by SI (dichotomized by the median SI), the probability of post-intubation hypotension was 24% (95% CI, 21–28%) in patients with a SI 0.90–1.12 and 21% (95% CI, 17–26%) in patients with a SI ≥ 1.13. In both groups, ketamine use was significantly associated with a lower risk of post-intubation hypotension compared with the reference group (adjusted odds ratio [aOR], 0.39 [95% CI, 0.23–0.65] in patients with a SI of 0.90–1.12; aOR 0.42 [95% CI, 0.23–0.76] in patients with a SI ≥ 1.13). Likewise, in the multivariable model including SI (as a continuous variable) and product term of ketamine use–X–SI, both SI and product term were not significantly associated with the risk of post-intubation hypotension, indicating that the effect of ketamine use on the risk of post-intubation hypotension does not differ by the degree of pre-intubation hemodynamic stability.

## Discussion

In this analysis of 977 adult hemodynamically-unstable patients who underwent intubation in the ED, we found that ketamine use as a sedative agent was associated with a significantly lower risk of post-intubation hypotension, compared with the reference group. This association remained significant across the use of different statistical assumptions—e.g., the analyses of multivariable hierarchical model and PS-matched model. To the best of our knowledge, this is the first study that has investigated the association between ketamine use and the risk of post-intubation hypotension in ED population.

These findings are consistent with the previous non-emergency-medicine literature that has reported the benefit of ketamine use for intubation. For example, a single-center randomized control trial of 80 patients in the operative room setting, White *et al*. have reported that thiopental decreased the mean arterial pressure by 11%, whereas ketamine increased the mean arterial pressure by 10%^6^. By contrast, several studies have also shown that the use of ketamine was associated with a higher risk of post-intubation hypotension in hemodynamically-unstable patients^7,8^. For example, a single-center descriptive study of 12 critically-ill operating room patients has reported that six patients had a decreased ventricular contractility and four patients had decreases in cardiac output or mean arterial blood pressure after ketamine use^7^. In addition, in an observational study of 120 out-of-hospital patients managed by a single emergency medical service, the patients with a high SI (≥0.9) had not only a blunted sympathetic response but also higher risk of post-intubation hypotension compared to patients with a low SI (<0.9)^8^. The reasons of these discrepancies between the studies might be attributable to the difference of study design (e.g., examination of the effect of ketamine according to SI), sample (e.g., differences in the proportion of the elderly), and study settings (e.g., operative room, out-of-hospital, and ED settings)^4,7–9^, or any combination of these factors. Nevertheless, the validity of our findings is buttressed by the use of data from a multi-ED prospective study with high quality data (e.g., 97% capture rate) with a sample size that is many times larger than any other prior studies in this topic as well as rigorous adjustment for potential confounding.

The underlying mechanisms of the favorable effect of ketamine on hemodynamically-unstable patients are likely multifactorial. In addition to its amnestic, analgesic, and bronchodilation properties, ketamine also releases catecholamines and increases norepinephrine levels by inhibiting their neuronal and extraneuronal reuptake, thereby increasing heart rate, cardiac output, and arterial pressure^4^. Dowdy *et al*. demonstrated that the cardio-stimulatory effects were attributable to enhanced norepinephrine release secondary to depressed baroreceptor reflex activity^10^. In addition, the literature has also suggested that the use of midazolam and propofol is associated with a higher risk of post-intubation hypotension^11^. Consequently, our findings may be explained, at least partially, by the combination of these potential mechanisms.

According to the World Health Organizations Report, ketamine is the most commonly-used anesthetic agents in the developing countries because ketamine is readily available and inexpensive^12^. While etomidate is frequently used for intubation in industrialized countries (e.g., North America), etomidate is not approved in many developing countries and some industrialized countries (e.g., Japan). A previous randomized controlled study comparing ketamine and etomidate in out-of-hospital and ED patients showed no significant difference in the severity of organ dysfunction and mortality after intubation^13^. In addition, while some controversy persists regarding the use of ketamine for rapid sequence intubation in patients with multisystem trauma at risk for both hypotension and elevated intracranial pressure^14^, the evidence to refute its use is not robust. Indeed, studies have indicated that ketamine does *not* interfere with cerebral metabolism, reduce regional glucose metabolism, or increase oxygen consumption *but* may benefit patients with a neurologic injury by a catecholamine-mediated increase in arterial pressure and cerebral perfusion^15–18^. Building on the currently-available evidence, our findings lend another significant support to the use ketamine for hemodynamically-unstable patients in the ED, particularly where etomidate is not available or approved for intubation^19^.

The present study has several potential limitations. First, the data do not include information on post-ED outcomes, such as in-hospital mortality. While post-intubation hypotension might be considered as a transient adverse event, the literature has documented that the post-intubation hypotension in the ED is a risk factor for higher in-hospital mortality and longer hospital length of stay^1,20^. Second, there may have been be a self-reporting bias, which may lead to an underestimation of the proportion of post-intubation hypotension. However, we used the previously applied standardized protocol with structured data forms and high capture rate^21–25^ and the incidence of post-intubation hypotension in our study was consistent to that was reported in a previous systematic review^26^. Third, despite the rigorous adjustment, the causal inference (i.e., the contribution of ketamine use to the risk of post-intubation hypotension) may have been confounded by unmeasured factors, such as infusion volume, vasopressor use, and comorbid illnesses (e.g., heart failure). Yet, the multivariable model adjusting for the primary indication and PS-matching addressed, at least partially, such unmeasured confounding. Lastly, our study sample chiefly consisted of patients in the academic EDs in Japan and hence our inferences may not be generalized in different practice settings. While it is tempting to dismiss the broader applicability of these findings, these are pharmacologically and clinically plausible, and are likely present in different clinical settings^4,5,7^.

Based on the analysis of data from a large prospective multicenter study of ED airway management, we found that the use of ketamine was associated with a significantly lower risk of post-intubation hypotension, compared to the use of midazolam or propofol, in hemodynamically-unstable ED patients. The significant association persisted across the different statistical assumptions. As post-intubation hypotension is an important risk factor of unfavorable post-ED outcomes^1,20^, for clinicians, our observations lend significant support to the use of ketamine as a sedative for rapid sequence intubation in hemodynamically-unstable patients. For researchers, our data should advance the research into the development of optimal airway management strategies, which will, in turn, improve outcomes in critically-ill patients in the ED.

## Methods

### Study design and setting

This is an analysis of the data from a multicenter prospective observational study of consecutive ED patients who underwent emergency airway management— JEAN-2 registry from February 2012 through November 2016. The study design, setting, methods of data collection, and measured variables have been reported elsewhere^21–25,27,28^. In brief, the JEAN-2 study is a consortium of 15 academic and community medical centers from different geographic regions across Japan. The participating institutions were certified as 12 level I and three level II equivalent trauma centers. These EDs had a median of 27,000 patient visits in the ED per year (range, 14,000–65,000). All institutions were affiliated with emergency medicine residency training programs and staffed by emergency medicine attending physicians. Transitional-year residents (postgraduate-years 1 and 2) also rotated through the ED and participated in intubations. Each ED maintained individual protocols about the policy and procedures for ED intubations. Intubations were performed by resident physicians or attending physicians at the discretion of attending physicians. The study was approved by the institutional review board of each participating hospital with waiver of informed consent.

### Selection of participants

In the present study, we selected all adult non-cardiac-arrest patients (aged ≥18 years) who underwent intubation in the ED with a pre-intubation SI of ≥0.9, as a marker of hemodynamic instability^8^. SI of ≥0.9 better represents hemodynamical instability compared with SBP alone^8^. We excluded (1) patients who received no sedatives, (2) patients who received sedatives other than ketamine, propofol, or midazolam (i.e., diazepam and thiopental that were used for 7% of patients with any sedative use in the study)^23^, (3) patients who received two or more types of sedatives, and (4) patients with missing data on age, weight, SBP or heart rate at pre-intubation, SBP at post-intubation, sedative, intubation device, or specialty of intubator.

### Data collection and processing

Immediately after each intubation encounter, the intubator completed a standardized data collection form that included the patient demographics (e.g., age, sex), weight and height, primary indication for the intubation, methods of intubation, all medications and devises used to facilitate airway management, level of training and specialty of the intubator, number of attempts, success or failure, associated adverse events, and vital signs (heart rate, blood pressure, arterial oxygen saturation) measured immediately before, immediately after, and 30 minutes after the intubation^19,21–25,27–30^. The JEMNet (Japanese Emergency Medicine Network) coordinating center and site investigator at each ED monitored compliance with data form completion. Where the data form was missing, it was returned to the intubator for completion. If information on the data form contained contradiction, the investigator interviewed the intubator for airway management detail. These *post hoc* interviews occurred within fourteen days of the patient encounter. An intubation “attempt” was defined by a single insertion of the laryngoscope (or other device) past the teeth. An attempt was recognized as success if tracheal tube was placed through the vocal cords and confirmed by a quantitative or colorimetric end-tidal carbon dioxide monitor.

### Primary exposure

The primary exposure of interest was ketamine use for emergency airway management, compared with midazolam or propofol use as the reference.

### Outcome measure

The outcome measure of interest was post-intubation hypotension, defined as SBP of ≤90 mmHg during the 30-minute period following intubation or ≥20% decrease in SBP between pre-intubation and immediately after intubation^3,19,24,29,31^.

### Statistical analysis

For the purpose of the present analysis, we categorized the patients into the ketamine and reference (i.e., midazolam or propofol use) groups. To investigate the association between the use of ketamine and the risk of post-intubation hypotension, we constructed unadjusted and adjusted two-level hierarchical models with a binomial response using random intercepts for the EDs to account for patient clustering within the ED. In the multivariable analysis, we adjusted for age, sex, body mass index (BMI), primary indication, premedication use, paralytic use, and intubator’s specialty. These covariates were chosen based on the clinical plausibility and *a priori* knowledge^19,27,29,32,33^. BMI was classified into three categories (<18.5, 18.5–24.9, and ≥25.0 kg/m^2^)^27^. Primary indication for intubation was classified into four categories: respiratory failure, medical shock, traumatic indication, and others (e.g., airway obstruction, altered mental status, other medical indications)^33^. Specialty of the intubator was categorized into transitional-year resident, emergency medicine resident, emergency attending physician, and other specialties.

In the sensitivity analysis to determine the robustness of our inference, we also performed a propensity matching (PS) analyses. First, we computed the PS by fitting a logistic regression model that estimates the probability of ketamine use conditional on the covariates in the primary multivariable model above. Second, we conducted one-to-one matching of patients between the ketamine and reference groups with the closest estimated PS within a caliper (≤0.20 of the pooled standard deviation of estimated logits) using the nearest neighbor method without replacement^34^. We computed the standard differences to examine the appropriate matching of the baseline characteristics between the two groups. A standard difference of >10% was regarded as imbalanced. Lastly, in the PS-matched patients, we conducted a logistic regression analysis to examine the association between ketamine use and risks of post-intubation hypotension.

To determine whether the effect of ketamine use on the risk of post-intubation hypotension differs by the degree of pre-intubation hemodynamic stability, we conducted two additional analyses. First, we stratified the patients into two groups according to the median SI: (1) patients with a SI of 0.90–1.12 and (2) patients with a SI of ≥1.13. Then, we fit the multivariable model for each group. Second, we also fit a multivariable model including a product term of ketamine use–X–SI. P < 0.05 was considered statistically significant. Analyses were performed with the use of STATA 14.1 (StataCorp, College Station, TX) and JMP 12.2.0 (SAS Institute, Inc., Cary, NC).

## Supplementary information

Supplementary Information

Supplementary Information

## Data Availability

The datasets generated during and/or analyzed during the current study are not publicly available due to no IRB’s approval of data sharing but are available from the corresponding author on reasonable request.
