# Author Correction: Association of ketamine use with lower risks of post-intubation hypotension in hemodynamically-unstable patients in the emergency department

**DOI:** 10.1038/s41598-020-58489-3

**Published:** 2020-02-05

**Authors:** Tadayoshi Ishimaru, Tadahiro Goto, Jin Takahashi, Hiroshi Okamoto, Yusuke Hagiwara, Hiroko Watase, Kohei Hasegawa, Japanese Emergency Medicine Network Investigators, Hiroshi Morita, Takahisa Kawano, Yohei Kamikawa, Hideya Nagai, Takashi Matsumoto, Suguru Nonami, Yusuke Miyoshi, Sho Segawa, Yuya Kitai, Kenzo Tanaka, Saburo Minami, Hiromasa Yakushiji, Hiroshi Okamoto, Naoto Miyauchi, Yukari Goto, Nobuhiro Sato, Koichiro Gibo, Masashi Okubo, Yukiko Nakayama, Nobuhiro Miyamae, Hirose Kaoru, Taichi Imamura, Azusa Uendan, Yasuaki Koyama, Hiroshi Kamura, Nakashima Yoshiyuki, Jin Takahashi, Jin Irie, Nobunaga Okada, Seiro Oya, Akihiko Inoue

**Affiliations:** 1 Department of Emergency and Critical Care Medicine, Tokyo Bay Urayasu Ichikawa Medical Center, 3-4-32 Todaijima, Urayasu, Chiba 279-0001 Japan; 2 Graduate School of Medical Sciences, University of Fukui, 23-3, Shimoaizuki, Matsuoka, Eiheiji, Yoshida, Fukui 910-1193 Japan; 3 Department of Critical Care Medicine, Kurashiki Central Hospital, 1-1-1 Miwa, Kurashiki, Okayama 710-8602 Japan; 4 Department of Pediatric Emergency and Critical Care Medicine, Tokyo Metropolitan Children’s Medical Center, 2-8-29 Musashidai, Fuchu, Tokyo 183-8561 Japan; 5 Department of Radiology, University of Washington, 850 Republican Street Seattle, Washington, WA 98006 USA; 6 Department of Emergency Medicine, Massachusetts General Hospital, Harvard Medical School, Boston, MA USA; 7 Fukui University Hospital, Fukui, Japan; 8 Fukui Prefectural Hospital, Fukui, Japan; 9 Kameda Medical Center, Chiba, Japan; 10 Kishiwada Tokushukai Hospital, Osaka, Japan; 11 Nagoya Ekisaikai Hospital, Nagoya, Japan; 12 Niigata City General Hospital, Niigata, Japan; 13 Okinawa Chubu Prefectural Hospital, Okinawa, Japan; 14 Otowa Hospital, Kyoto, Japan; 15 Shonan Kamakura General Hospital, Kanagawa, Japan; 16 St. Marianna University School of Medicine Hospital, Kanagawa, Japan; 17 Tokyo Bay Urayasu Ichikawa Medical Center, Chiba, Japan; 18 University Hospital, Kyoto Prefectural University of Medicine, Kyoto, Japan; 19 Yokohama Rosai Hospital, Kanagawa, Japan; 20 Hyogo Emergency Medical Center, Hyogo, Japan

Correction to: *Scientific Reports* 10.1038/s41598-019-53360-6, published online 21 November 2019

The original version of this Article contained a typographical error in the spelling of the consortium Japanese Emergency Medicine Network Investigators which was incorrectly given as Emergency Network Investigators. This has now been corrected in the PDF and HTML versions of the Article.

