# Supplementary material for: Association of ketamine use with lower risks of post-intubation hypotension in hemodynamically-unstable patients in the emergency department
Source: Sci Rep. 2019 Nov 21;9:17230. doi: 10.1038/s41598-019-53360-6 (PMC6872717; doi:10.1038/s41598-019-53360-6)
Supplement: Supplementary file 1 — Supplementary Information [file 41598_2019_53360_MOESM1_ESM.pdf]

Supplemental Table 1. Baseline characteristics of the original and propensity score-matched cohorts

	**Original cohort**			**PS matched cohort**		
**Variables**	Ketamine group (n=316)	Reference group （n=661）	Standardized differences‖	Ketamine group (n=286)	Reference group （n=286）	Standardized differences‖
Age, mean (SD), years	65.6 (17.0)	63.7 (17.7)		64.5 (16.1)	63.8 (18.1)	
Female sex (%)	209 (66.1)	460 (69.6)	7.4	65.8 (17.0)	65.0 (16.5)	1.3
Body mass index (kg/m^2)						
<18.5	63 (19.9)	111 (16.8)	8.0	49 (17.1)	48 (16.8)	0.8
18.5-24.9	169 (53.5)	398 (60.2)	13.6	165 (57.7)	167 (58.4)	1.4
≥25.0	84 (26.7)	152 (23.0)	8.5	72 (25.2)	71 (24.8)	0.9
Primary indication (%)						
Respiratory failure	75 (23.7)	246 (37.2)	29.7	75 (26.2)	73 (25.5)	1.6
Medical shock	166 (52.5)	144 (21.8)	67.0	136 (47.6)	134 (46.9)	1.4
Traumatic indication	40 (12.9)	98 (14.8)	55.0	40 (14.0)	45 (15.7)	4.8
Others*	35 (11.1)	173 (26.2)	39.5	35 (12.2)	34 (11.9)	0.9
Pretreatment use	216 (32.7)	445 (67.3)	73.8	99 (34.6)	92 (32.2)	5.1
Neuromuscular blocker use†	254 (80.4)	487 (73.7)	16.0	226 (79.0)	226 (79.0)	0
Intubator's specialty						
Transitional-year resident‡	92 (29.1)	244 (36.9)	16.6	90 (31.5)	91 (31.8)	6.5
Emergency medicine resident	136 (43.0)	224 (33.9)	18.8	114 (39.9)	119 (41.6)	3.5
Emergency physician	51 (16.1)	121 (18.3)	5.8	50 (17.5)	49 (17.1)	1.1
Other specialties	37 (11.7)	72 (19.9)	22.6	32 (11.2)	27 (9.4)	5.9
Outcome Event						
Post-intubation hypotension§	47 (14.9)	180 (27.2)	30.5	45 (15.7)	81 (28.3)	30.8

Abbreviations: SD, standard derivation; PS, propensity score.

* Defined as airway obstruction, altered mental status, and other medical indications

† With or without succinylcholine, rocuronium, or vecuronium.

‡ Defined as post-graduate years 1 or 2.

§ Systolic blood pressure of ≤90 mmHg during the 30-minute period following intubation or ≥20% decrease in systolic blood pressure between pre-intubation and immediately after intubation.

|| Standardized difference of >10 indicates significant imbalance of a baseline covariate.
