# Supplementary figures and images for: Association of ketamine use with lower risks of post-intubation hypotension in hemodynamically-unstable patients in the emergency department
Source: Sci Rep. 2019 Nov 21;9:17230. doi: 10.1038/s41598-019-53360-6 (PMC6872717; doi:10.1038/s41598-019-53360-6)

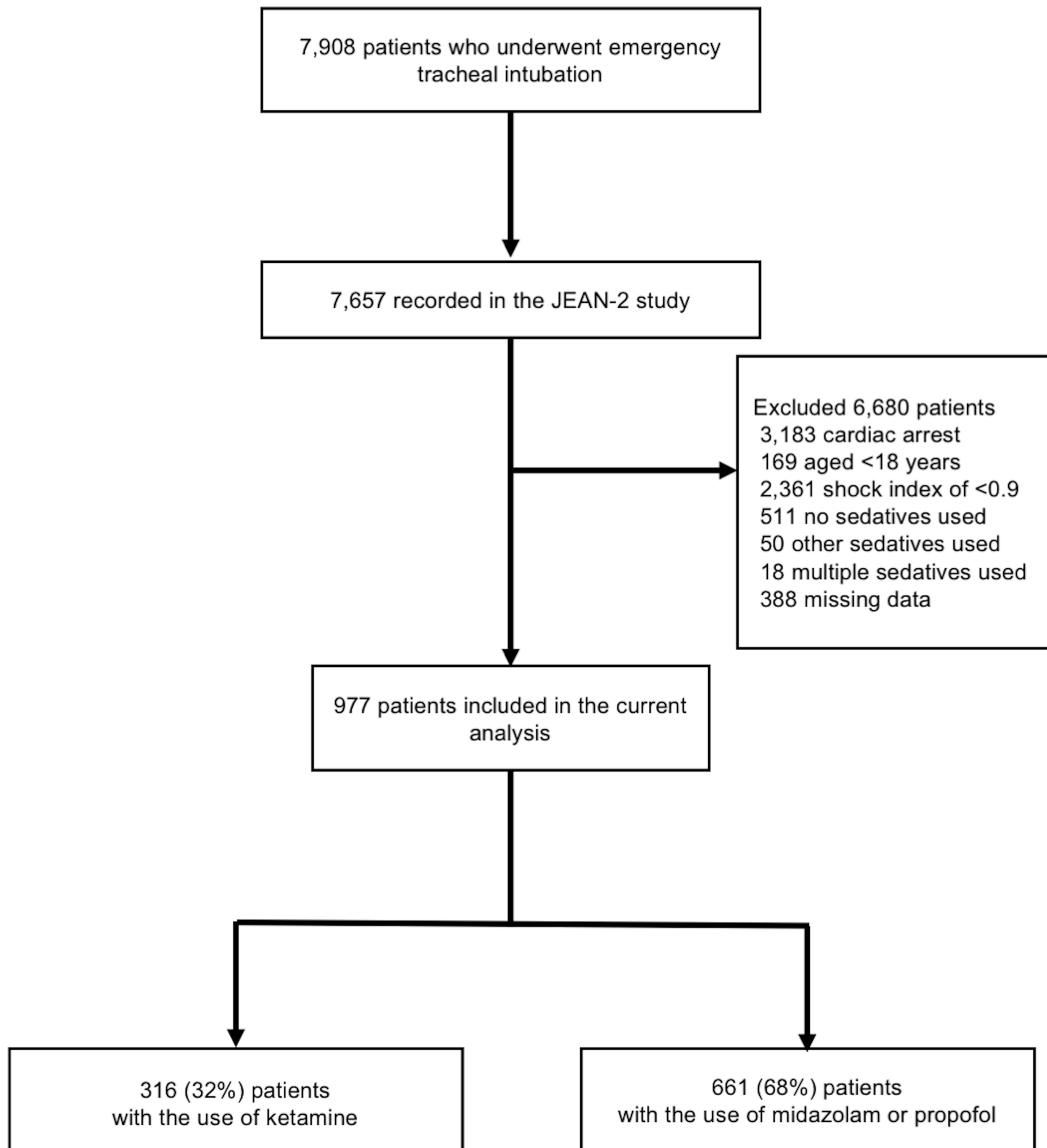

Supplement: Supplementary file 2 — Supplementary Information [file 41598_2019_53360_MOESM2_ESM.pdf]
